# Clinical presentation and hematological profile among young and old chronic lymphocytic leukemia patients in Sudan

**DOI:** 10.1186/s13104-019-4239-7

**Published:** 2019-04-02

**Authors:** Ameen Abdulaziz Basabaeen, Enaam Abdelrhman Abdelgader, Ebtihal Ahmed Babekir, Nada Hassan Eltayeb, Osama Ali Altayeb, Eman Abbass Fadul, Othman Saeed Bahashwan, Ibrahim Khider Ibrahim

**Affiliations:** 1 Department of Hematology, Faculty of Medical Laboratory Sciences, Al Neelain University, Khartoum, Sudan; 2 Department of Pathology, Faculty of Medicine, Al Neelain University, Khartoum, Sudan; 3 Flow Cytometry for Leukemia & Lymphoma Diagnosis, Khartoum, Sudan; 4 Department of Family Medicine, Faculty of Medicine, University of Gezira, Wad Medani, Sudan; 5 Department of Physiology, Faculty of Medicine, Al Neelain University, Khartoum, Sudan

**Keywords:** CLL, Sudan, Clinical, Hematological, Staging, Rai, Binet, Young, Old

## Abstract

**Objective:**

To assess the clinical presentation and hematological profile among young (≤ 55 years) and old (> 55 years) chronic lymphocytic leukemia patients in Sudan.

**Result:**

In the present cross-sectional descriptive study, out of 110 cases studied, among them 31 (28.2%) were young (≤ 55 years) patients with mean age 48 years, and 79 (71.8%) were elder patients (> 55 years) with mean age 66 years, the overall mean age was 62.97 ± 12.06 with range (22–85 years), and 79 (71.8%) were males and 31 (28.2%) were females (M:F = 2.6:1) (P = 0.000). (7.3%) were asymptomatic, 61 (55.5%) presented with nonspecific complains. Generalized lymphadenopathy was seen in 52 (47.27%) with elder predominance (P = 0.03). Splenomegaly, hepatomegaly, thrombocytopenia and anemia were seen in 54 (49.1%), 14 (12.7%), 43 (39.1%) and 38 (34.5%) of patients respectively with male predominance. 54 (49.1%) and 42 (38.18%) of patients presented at Rai high risk and Binet C stages respectively with nearly same age and sex distribution. CLL in Sudan is a disease of elders, same as seen in literature, with high male to female ratio. In general hematological parameters means were noted to be distributed equally according to age and sex groups. Majority of patients were presented with nonspecific symptoms and nearly half of patients presented at late stages as reported in most developing countries.

**Electronic supplementary material:**

The online version of this article (10.1186/s13104-019-4239-7) contains supplementary material, which is available to authorized users.

## Introduction

Chronic lymphocytic leukemia (CLL) is characterized by the accumulation of mature-appearing lymphocytes in the blood, marrow, lymph nodes, and spleen [1, 2]. CLL diagnosis requires the presence of at least 5000 circulating B cells/µl with clonality demonstrated by flow cytometry according to International Workshop on Chronic Lymphocytic Leukemia (IWCLL) criteria [1]. CLL cells are monoclonal B lymphocytes that express CD19, CD5, and CD23 with weak or no expression of surface immunoglobulin (Ig), CD20, CD79b, and FMC7 [1, 2]. CLL is the most common leukemia of adults in the west countries, with an incidence of up to 50 cases per 100,000 persons older than 80 years of age [3, 4]. In contrast to the high prevalence observed in the West, CLL is much less common in some other parts of the world, notably Japan and China [5, 6]. Most CLL patients in the general population are elderly (median age 71.5 years). As a result of referral bias the median age of patients seen in the specialist clinic is 64 years, with 20% to 25% of patients being < 55 years old [7, 8]. The median age at diagnosis is younger for males (70 years) than for females (73 years), with the male: female ratio being 1.3:1 [9]. Nowadays, 70% to 80% of patients are diagnosed incidentally when they have a routine blood count and will have early-stage (Rai 0 or I) disease [10]. The natural history of CLL is extremely variable with survival times ranging from 2 to 20 years [11]. Overall, the response rate to therapy and survival is better in women than in men [12]. Patients with atypical morphologic and/or immunophenotypic features tend to have a more aggressive clinical course. In general, presence and extent of Lymphadenopathy, Splenomegaly, Hepatomegaly, anemia, and thrombocytopenia are the major clinical parameters that correlate with prognosis [13] Two staging systems are in general use, and are based on physical examination and results of a routine blood count [14, 15]. The Rai [14] and Binet [15] staging systems have been used for a long time for patients with CLL, and still remain relevant and complementary to molecular testing in the modern era [16]. However, the presenting features are similar regardless of age [8, 17]. Rai staging system categorizes CLL patients into 5 groups, with a simplified 3-stage version of this system now generally accepted [14, 18]. The normally adopted limit for the inclusion of patients into intensive chemotherapeutic regimes is 55 years [8, 19]. To the best of our knowledge this is the first study conducted to in Sudan to investigate the clinicohematological characteristics of CLL patients and staging classification according to the previous cut-off age groups. The objective of this study was to characterize the clinical and hematological features of young (≤ 55 years) and elderly (> 55 years) CLL patients in Sudan with sex variations.

## Main text

### Methods

This study was a cross-sectional descriptive study, conducted in Khartoum state, Sudan, in the period from April 2017 to April 2018. A total of 110 blood samples were collected from patients with Chronic Lymphocytic Leukemia in EDTA containing tube. Patients were obtained at Flowcytometry Laboratory Center Khartoum, Sudan, where the patients were referred for immunophenotypic diagnosis.

All patients were diagnosed on the basis of clinical history and physical examination, complete blood count, and immunophenotypic criteria [9]. The stage of the chronic lymphocytic leukemia was assessed by Binet and Rai [14, 15] classification. All patients were newly diagnosed without any previous CLL treatment.

#### Determination of immunophenotyping and blood count

The diagnosis of CLL was confirmed for each patient by Flowcytometry (EPICS XL Beckman Coulter flow cytometer, Miami, FL, USA), standard protocol of Beckman Coulter [20] was used in fluorescent dye labeled monoclonal antibody for CD5, CD10, CD19, CD20, CD22, CD23, FMC7, and diagnostic score was assessed by Matutes et al. [21], Absolute B Lymphocyte count obtained by Flowcytometry and Complete Blood Count, was performed using automated hematology analyzer (SYSMEX KX-21N, Japan), Total WBC, Absolute Lymphocyte Count, Hemoglobin level, RBC and Platelets were recorded. ZAP-70 and CD38 were used as prognostic markers, with a cutoff point of 20% and 30%, respectively.

#### Statistical analysis

Patient’s data was collected by structural interview questionnaire and from patient’s medical records and analyzed using the statistical package for social sciences (SPSS), version-23. Analysis was done for correlation between clinical and hematological variables and Chronic Lymphocytic Leukemia stages to compare means according to age and gender with variance of means using *T* test and ANOVA, and Kruskal–Wallis test and correlations with Pearson and Spearman.

### Results

**Fig. 1:**
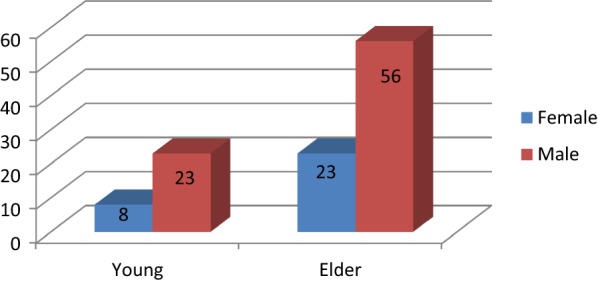
Gender Distribution within age groups

Out of 110 cases studied 31 (28.2%) were young (≤ 55 years) patients with mean age 48 years, and 79 (71.8%) were elder patients (> 55 years) with mean age 66 years (P = 0.000), the overall mean age was 62.97 ± 12.061 with range (22–85 years), and 79 (71.8%) were males and 31 (28.2%) were females (M: F = 2.6:1) (P = 0.000) (see Fig. 1).

4 patients (7.3%) were asymptomatic discovered during routine examination, and 61 (55.5%) presented with non specific complains (see Additional file 1: Figure S1). Fever (23.6%) was the most presenting complain in the rest (42.3% Young and 57.7% Elder, P = 0.099) (see Additional file 1: Figure S1). Lymphadenopathy was the most presenting sign seen in 78 (70.9%), of them (67.9%) were elder patients with male predominance (P = 0.005, OR = 3.42), and generalized lymphadenopathy was seen in 52 (47.3%) (see Additional file 2: Figure S2). Splenomegaly was seen in 54 (49.1%) patients, of them (64.5%) were young patients, and was negatively correlated to age in age groups (P = 0.044). Splenomegaly was significantly correlated with lymphadenopathy, hepatomegaly, thrombocytopenia, but not anemia (P = 0.049, 0.003, 0.001, 0.182 respectively). Hepatomegaly was seen in 14 (12.7%), and half of them were elder male. Hepatosplenomegaly was seen in 12 (10.9%), 25% of them were young and 75% elder (P = 0.796) (see Table 1). TWBCs was inversely correlated to age (P = 0.187), with highest mean in young males (113.88 ± 108.45) and lowest in elder females (72.68 ± 57.55) (P = 0.183). Highest Means for absolute lymphocyte and Monoclonal B Lymphocytes count were seen in young males (101.21 ± 101.72, 91.13 ± 96.78, respectively) and lowest in elder females (63.33 ± 51.73, 55.94 ± 48.52, respectively), with no significant correlation found with age and sex groups (see Table 2). Thrombocytopenia was seen in 43 (39.1%). Anemia was seen in 38 (34.5%) of patients, 17 of them were elder males. Fifty-five (49.1%) of patients presented at advanced Rai stage (III, IV) with nearly equal distribution in both age groups, followed by 46 (41.82%) presented at intermediate stage (I, II), and the rest 10 (9.1%) at stage (0) with 90% of them were elder. The only significant correlation for gender with Rai staging was for Rai stage (0) (P = 0.016). Forty-two (38.18%) of patients presented in Binet stage C, of them 29 (36.71%) were elders.

**Table 1:** Clinical characteristics according to age group and sex

Parameter	≤ 55 years, n = 31	> 55 years, n = 79	P value*	OR	Male, n = 79	Female, n = 31	P value	OR
Generalized lymphadenopathy	20	32	0.03*	0.37	41	11	0.14	0.51
Splenomegaly	20	34	0.044*	0.41	41	13	0.352	0.67
Hepatomegaly	3	11	0.402	1.50	9	5	0.357	1.50
Hepatosplenomegaly	3	9	0.548	1.20	8	4	0.452	1.31
Thrombocytopenia	17	26	0.34	0.40	31	12	0.960	0.98
Anemia	12	26	0.569	0.78	25	13	0.312	1.56

n = 110

* P value significant below 0.05; Kruskal–Wallis test

**Table 2:** Hematological characteristics of age groups

Parameter	≤ 55 years, n = 31 (28.18%)	> 55 years, n = 79 (71.82%)	P value*
Mean age years (range)	48 (22–55)	69 (56–85)	
Sex (male)	23 (74.2%)	56 (71.8%)	0.730
Mean white cell count (× 10^3^/μl)	108.07 ± 96.54 (9.7–350.7)	86.49 ± 65.12 (11.9–293.0)	0.187
Mean absolute lymphocyte count (× 10^3^/μl)	96 ± 90.42 (7.76–325.5)	76.71 ± 61.47 (9.52–281.28)	0.200
Mean absolute B-cell count (× 10^3^/μl)	86.07 ± 86.09 (6.70–302.39)	68.23 ± 57.57 (7.61–274.25)	0.210
Mean hemoglobin (g/dl)	11.13 ± 2.78 (4.5–15.6)	11.16 ± 2.35 (4.40–18.10)	0.956
Mean platelets count (× 10^3^/μl)	166.47 ± 78.06 (13.70–356)	198.18 ± 112.92 (29–587)	0.155
Mean red cell count (× 10^3^/μl)	3.69 ± 1.08 (1.19–5.43)	3.71 ± 0.89 (1.98–6.18)	0.897

n = 110

* P value significant at 0.05, independent T test (2-tailed)

ZAP-70 and CD38 were positively expressed in 11 (35.5%) and 14 (45.2%) of young patients, and in 25 (31.6%) and 27 (34.2%) of elder patients, respectively.

### Discussion

CLL is the most common form of adult leukemia in the Western countries, with most of cases in elders above 50. In Sudan few data available about accurate prevalence and all research done yet was obtained at the same center (Khartoum Radioisotope Center, Khartoum, Sudan), and most cases diagnosed at advanced stages due to insufficient diagnostic facilities and lack of appropriate health education. In this study (71.8%) of CLL patients were elder above 55 years, with mean age was 62.97 ± 12.061, similar results were reported by Ahmed and Osman [22] and Salawu et al. [23] in Africa, Seftel et al. and Mauro et al. [8, 9] in western countries, and Zeeshan et al., Agrawal et al. [24, 25] in Asia. In this study the gender distribution was similar in CLL young patients ≤ 55 years and elder > 55 years, whereas a male predominance had been noted by Maruro et al. and Pamuk et al. [8, 26]. In all CLL patients, male to female ratio was (2.6:1) which was higher than that in western countries (1.3:1) Seftel et al. [9], and that reported in Sudan (1.2:1) by Elsukker et al. [27], and in contrast with the result (0.8:1) obtained by Salawu et al. [23] in Nigeria. Higher male to female ratio in this study may be due to more exposure to environmental and occupational hazards in males than females, which is in consistent with those reported by Agrawal et al. [25], Ahmed and Osman [22] and Ahmed et al. [28]. Accidental diagnosis of CLL was reported in 30–35% of cases according to literature, however this study showed only (7.3%) were accidentally diagnosed, similar low percentage shown by Ahmed and Osman. [22] in Sudan, mostly due to presentation at late stages of disease and deficits in referring system and loss of behavior of regular medical checkup [11]. In this study lymphadenopathy was the most presenting finding (70.9%), most of them were elder males, higher than results recorded by Agrawal et al. [25] and Sulhyan et al. [29] (55% and 46.15%, respectively), but in consistent with higher results of Ahmed and Osman [22] in Sudan. Late stage presentation in Sudan reflects this higher results and may be ethnic heterogeneity [30].

Splenomegaly was seen in 54 (49.1%) in our patients and presented more at elder group with male predominance, which agree with Zeeshan et al. [24] results (46.6%), but lower than results by Agrawal et al. [25], Salawu et al. [23] and Elsukker et al. [27], (66%), (70.9%) and (86%) respectively. In this study splenomegaly was correlated with lymphadenopathy, hepatomegaly, thrombocytopenia, but not anemia (P value = 0.048, 0.003, 0.000, and 0.182 respectively). Hepatomegaly was seen in 14 (12.7%) of our patients and half of them were elder males, whereas Agrawal et al. [25] and Gogia et al. [31] reported a higher percentage (63%) and (40%) in contrast to Ahmed and Osman [22] and Rozman et al. [32] who reported only (3.1%) and (10%) respectively. Hepatosplenomegaly was seen in 12 (%) and 75% of them were elder group patients. Highest TWBCs mean was seen in young males (113.40 ± 107.40) and lowest in elder females (72.68 ± 57.55). Absolute lymphocytes count and Monoclonal B lymphocytes were similarly distributed in age and sex groups with no significant correlation in age and sex groups (see Additional file 3: Table S1). Of our patients, only 10 (9.1%) presented in Rai stage (0) and the highest percentage group was stage (III) 36 (32.7%), followed by (I) and (II) 23 (20.9%) for both, and (IV) 18 (16.4%) (see Additional file 4: Table S2). Using modified Rai staging, 54 (49.1%) of our patients were at high risk stage (III + IV), 46 (41.8%) at intermediate stage (I + II) and only 10 (9.1%) at low risk stage (see Additional file 5: Table S3). Frequencies in age groups for Binet stage A, B, and C were 33 (30%), 35 (31.8%), and 42 (38.2%), respectively (Data was shown in Additional file 6: Table S4). Rai and Binet stages distributions according to sex are shown in Additional file 7: Figures S3 and Additional file 8: Figure S4. No significant correlation with age groups in both staging scores and the significant correlation with sex was seen between Rai 0 and other modified Rai stages (P = 0.016, and 0.48). Similar trends for Rai and Binet stages were reported by Gogia et al. [31] in India and Ahmed and Osman [22] in Sudan. Unsurprisingly reverse patterns with highest patients percentages at stage (Rai 0 and Binet A) and lowest percentages at advanced stages (III, IV, and C) in developed countries were reported by Mauro et al. [8] in Italy and Apelgen et al. [33] in Sweden. This contrast patterns may be explained by the gap in advances of health infrastructures and management seeking behavior between developed and developing countries and because more patients in developing countries presented in late stages. Considering ZAP-70, when 20% used as cutoff for positivity, 36/110 (32.7%) of our patients had positive ZAP-70 expression and regarding CD38 expression by using 30% cutoff, 41/110 (37.3%) were positive. Analysis of ZAP-70 and CD38 showed no significant difference in expression between age groups (see Additional file 9: Table S5). Our results agree with, Xu et al. [34] who failed to find statistically significant difference between the expression of the CD38 and ZAP-70 in young versus elder patients. Also agree with Parikh et al. [35] who reported same conclusion for CD38 expression and gender. This study disagrees with Parikh et al. [35] who reported significant difference in ZAP-70 expression between young and elder patients. Conclusion: Our study concluded that CLL in Sudan is a disease of elders, with mean age was 63 years and range (22–85 years), same as seen in literature, with male to female ratio (2.6:1). In general Hematological parameters means were noted to be distributed equally according to age and sex groups and no significant differences in expression of ZAP-70 and CD8 in young versus elder patients and gender. Majority of patients were presented with non specific symptoms. Lymphadenopathy and Splenomegaly were seen in most elder males. (49.1%) and (38.2%) of patients were at Rai high risk stage and Binet C stage, respectively at presentation. Further extensive multicenter studies which include genetic markers, bone marrow infiltration, occupational effect, and tribal variation in Sudan may yield a clearer image for CLL in Sudan.

## Limitations

Limitations which are worth to mention are: sampling method was depended on voluntary participation and no bone marrow samples were obtained, patients were not followed up for progression of B-CLL, survival rates and response to treatment administered after diagnosis confirmation, No radiological confirmation or previous radiological data evaluated, and reticulocyte count and Coomb’s test was not done for co-existence of autoimmune disease which may affect study results. The previous limitations should be considered in interpretation of this study results.

## Additional files

**Additional file 1: Figure S1.** Frequencies of the most common symptoms in our patients.

**Additional file 2: Figure S2.** Distribution of generalized lymphadenopathy by age groups.

**Additional file 3: Table S1.** Hematological characteristics according to sex.

**Additional file 4: Table S2.** Rai stage in age groups.

**Additional file 5: Table S3.** Modified Rai stage in age groups.

**Additional file 6: Table S4.** Binet stage in age groups.

**Additional file 7: Figure S3.** Modified Rai risk staging with sex.

**Additional file 8: Figure S4.** Binet staging with sex.

**Additional file 9: Table S5.** Expression of CD38 and ZAP70 according to age and sex groups.

## Abbreviations

CLL: chronic lymphocytic leukemia
IWCLL: International Workshop of Chronic Lymphocytic Leukemia
CD: cluster of differentiation
EDTA: ethylene di amine tetra acetic acid
SPSS: Statistical Package for Social Sciences
WBC: white blood cell
RBC: red blood cell

## References

[1] Hallek M, Cheson BD, Catovsky D, Caligaris-Cappio F, Dighiero G, Döhner H, Hillmen P, Keating MJ, Montserrat E, Rai KR (2008). Guidelines for the diagnosis and treatment of chronic lymphocytic leukemia: a report from the International Workshop on Chronic Lymphocytic Leukemia updating the National Cancer Institute-Working Group 1996 guidelines. Blood.

[2] Landgren O, Albitar M, Ma W, Abbasi F, Hayes RB, Ghia P, Marti GE, Caporaso NE (2009). B-cell clones as early markers for chronic lymphocytic leukemia. N Engl J Med.

[3] O’Brien S, Del Giglio A, Keating M (1995). Advances in the biology and treatment of B-cell chronic lymphocytic leukemia. Blood.

[4] Linet M, Blattner W, Polliack A, Chur CD (1988). Epidemiology of chronic lymphocytic. Chronic lymphocytic leukemia.

[5] Chan L, Lam C, Yeung T, Chu R, Ng M, Chow E, Wickham N, Matutes E (1997). The spectrum of chronic lymphoproliferative disorders in Hong Kong. A prospective study. Leukemia.

[6] Crowther-Swanepoel D, Houlston RS (2010). Genetic variation and risk of chronic lymphocytic leukaemia. Seminars in cancer biology.

[7] Yoon J-Y, Lafarge S, Dawe D, Lakhi S, Kumar R, Morales C, Marshall A, Gibson SB, Johnston JB (2012). Association of interleukin-6 and interleukin-8 with poor prognosis in elderly patients with chronic lymphocytic leukemia. Leukemia Lymphoma.

[8] Mauro FR, Foa R, Giannarelli D, Cordone I, Crescenzi S, Pescarmona E, Sala R, Cerretti R, Mandelli F (1999). Clinical characteristics and outcome of young chronic lymphocytic leukemia patients: a single institution study of 204 cases. Blood.

[9] Seftel M, Demers A, Banerji V, Gibson S, Morales C, Musto G, Pitz M, Johnston J (2009). High incidence of chronic lymphocytic leukemia (CLL) diagnosed by immunophenotyping: a population-based Canadian cohort. Leuk Res.

[10] Shanafelt TD, Byrd JC, Call TG, Zent CS, Kay NE (2006). Narrative review: initial management of newly diagnosed, early-stage chronic lymphocytic leukemia. Ann Intern Med.

[11] Rai KR, Keating MJ (2006). Clinical manifestation and diagnosis of chronic lymphocytic leukemia. UpToDate..

[12] Catovsky D, Fooks J, Richards S (1989). Prognostic factors in chronic lymphocytic leukaemia: the importance of age, sex and response to treatment in survival: a report from the MRC CLL 1 trial. Br J Haematol.

[13] Naeim F, Rao PN, Grody WW (2009). Hematopathology: morphology, immunophenotype, cytogenetics, and molecular approaches.

[14] Rai KR, Sawitsky A, Cronkite EP, Chanana AD, Levy RN, Pasternack B (1975). Clinical staging of chronic lymphocytic leukemia. Blood.

[15] Binet J, Auquier A, Dighiero G, Chastang C, Piguet H, Goasguen J, Vaugier G, Potron G, Colona P, Oberling F (1981). A new prognostic classification of chronic lymphocytic leukemia derived from a multivariate survival analysis. Cancer.

[16] Vasconcelos Y, Davi F, Levy V, Oppezzo P, Magnac C, Michel A, Yamamoto M, Pritsch O, Merle-Béral H, Maloum K (2003). Binet’s staging system and VH genes are independent but complementary prognostic indicators in chronic lymphocytic leukemia. J Clin Oncol.

[17] O’Brien S, Lerner S, Keating M: Chronic lymphocytic leukemia in the young patient. In: Seminars in oncology. 1998. p. 107–16. 9482532

[18] Rai KR, Han T (1990). Prognostic factors and clinical staging in chronic lymphocytic leukemia. Hematol Oncol Clin.

[19] Molica S, Brugiatelli M, Callea V, Morabito F, Levato D, Nobile F, Alberti A (1994). Comparison of younger versus older B-cell chronic lymphocytic leukemia patients for clinical presentation and prognosis. A retrospective study of 53 cases. Eur J Haematol.

[20] Beckman Coulter I. COULTER^®^ EPICS^®^ XL™ Flow Cytometer. 2010.

[21] Matutes E, Owusu-Ankomah K, Morilla R, Garcia Marco J, Houlihan A, Que T, Catovsky D (1994). The immunological profile of B-cell disorders and proposal of a scoring system for the diagnosis of CLL. Leukemia.

[22] Ahmed RAEM, Osman IM (2017). Clinical and haematological pattern of chronic lymphocytic leukaemia in sudanese patients. Int Blood Res Rev.

[23] Salawu L, Bolarinwa RA, Durosinmi MA (2010). Chronic lymphocytic leukaemia: a-twenty-years experience and problems in Ile-Ife, South-Western Nigeria. Afr Health Sci.

[24] Zeeshan R, Sultan S, Irfan SM, Kakar J, Hameed MA (2015). Clinico-hematological profile of patients with B-chronic lymphoid leukemia in Pakistan. Asian Pac J Cancer Prev.

[25] Agrawal N, Naithani R, Mahapatra M, Panigrahi I, Kumar R, Pati HP, Saxena R, Choudhary VP (2007). Chronic lymphocytic leukemia in India—a clinico-hematological profile. Hematology (Amsterdam, Netherlands).

[26] Pamuk GE, Pamuk ÖN, Soysal T, Öngören Ş, Başlar Z, Ferhanoğlu B, Aydin Y, Ülkü B, Aktuğlu G, Akman N (2001). An overview of young CLL patients: a single-centre experience from Turkey. Haematologia.

[27] Elsukker IHA, Eldirdiri MA, Enaam A, Abdelgadir SE, Abdalla IMO, Amira AK, Humeida M, Alya AS, Musa OH (2015). Clinical presentation and complications of chronic lymphocytic leukemia among Sudanese patients. Can Open Cancer J.

[28] Ahmed AMB, Yagoub TE, AlSaid AM, Mohammed JS, Osman SM (2016). Chronic leukemia in patients presenting to radiation and isotopes center–Khartoum during period from 1/2006 to 1/2007. Microscope.

[29] Sulhyan KR, Momin YA, Ratunavar PP, Gosavi SS, Patil DH (2017). Clinicopathological profile of patients with chronic leukaemia. Int J Health Sci Res.

[30] Gunawardana C, Austen B, Powell JE, Fegan C, Wandroo F, Jacobs A, Pratt G, Moss P (2008). South Asian chronic lymphocytic leukaemia patients have more rapid disease progression in comparison to White patients. Br J Haematol.

[31] Gogia A, Sharma A, Raina V, Kumar L, Vishnubhatla S, Gupta R, Kumar R (2012). Assessment of 285 cases of chronic lymphocytic leukemia seen at single large tertiary center in Northern India. Leuk Lymphoma.

[32] Rozman C, Bosch F, Montserrat E (1997). Chronic lymphocytic leukemia: a changing natural history?. Leukemia.

[33] Apelgren P, Hasselblom S, Werlenius O, Nilsson-Ehle H, Andersson PO (2006). Evaluation of clinical staging in chronic lymphocytic leukemia- population-based study. Leuk Lymphoma.

[34] Xu Z, Zhang J, Wu S, Zheng Z, Chen Z, Zhan R (2013). Younger patients with chronic lymphocytic leukemia benefit from rituximab treatment: a single center study in China. Oncol Lett.

[35] Parikh SA, Rabe KG, Kay NE, Call TG, Ding W, Schwager SM, Bowen DA, Conte M, Jelinek DF, Slager SL (2014). Chronic lymphocytic leukemia in young (≤ 55 years) patients: a comprehensive analysis of prognostic factors and outcomes. Haematologica.

