# Supplementary material for: Clinical presentation and hematological profile among young and old chronic lymphocytic leukemia patients in Sudan
Source: BMC Res Notes. 2019 Apr 2;12:202. doi: 10.1186/s13104-019-4239-7 (PMC6446286; doi:10.1186/s13104-019-4239-7)
Supplement: Supplementary file 1 — Additional file 1: Figure S1. Frequencies of the most common symptoms in our patients. [file 13104_2019_4239_MOESM1_ESM.docx]

Figure S1: Symptoms Distribution

Most patients presented with non specific symptoms and in the rest fever was the most presenting symptom.
