# Supplementary material for: Clinical presentation and hematological profile among young and old chronic lymphocytic leukemia patients in Sudan
Source: BMC Res Notes. 2019 Apr 2;12:202. doi: 10.1186/s13104-019-4239-7 (PMC6446286; doi:10.1186/s13104-019-4239-7)
Supplement: Supplementary file 2 — Additional file 2: Figure S2. Distribution of generalized lymphadenopathy by age groups. [file 13104_2019_4239_MOESM2_ESM.docx]

Figure S2: Distribution of generalized lymphadenopathy by age groups (n=110).

Percentage of young patients with generalized lymphadenopathy was 64.5%, comparing to only 40.5% of elders.
