# Supplementary material for: Clinical presentation and hematological profile among young and old chronic lymphocytic leukemia patients in Sudan
Source: BMC Res Notes. 2019 Apr 2;12:202. doi: 10.1186/s13104-019-4239-7 (PMC6446286; doi:10.1186/s13104-019-4239-7)
Supplement: Supplementary file 3 — Additional file 3: Table S1. Hematological characteristics according to sex. [file 13104_2019_4239_MOESM3_ESM.docx]

Table S1: Hematological characteristics according to sex

| Parameter | | N | Mean | Std. Deviation | Std. Error Mean | P value* |
| --- | --- | --- | --- | --- | --- | --- |
| TWBCs(x10^3^/ul) | Male | 79 | 98.89 | 81.025 | 9.116 | 0.182 |
|  | Female | 31 | 77.50 | 55.602 | 9.986 |  |
| RBCs(x10^6^/ul) | Male | 79 | 3.73 | .987 | .11108 | 0.646 |
|  | Female | 31 | 3.64 | .832 | .14950 |  |
| Platelets(x10^3^/ul) | Male | 79 | 186.76 | 104.853 | 11.797 | 0.693 |
|  | Female | 31 | 195.58 | 106.54 | 19.135 |  |
| HB(g/dl) | Male | 79 | 11.29 | 2.596 | .292 | 0.350 |
|  | Female | 31 | 10.80 | 2.105 | .378 |  |
| Absolute Lymphocytes  (x10^3^/ul) | Male | 79 | 87.74 | 77.100 | 8.674 | 0.189 |
|  | Female | 31 | 67.936 | 50.326 | 9.039 |  |
| Monoclonal B Lymphocytes  (x10^3^/ul) | Male | 79 | 78.49 | 72.772 | 8.187 | 0.193 |
|  | Female | 31 | 77.50 | 47.33 | 8.500 |  |

(n=110).*P value significant below 0.05, Independent T test (2-tailed)

Hematological parameters were comparable in male and female participants.
