# Supplementary material for: Clinical presentation and hematological profile among young and old chronic lymphocytic leukemia patients in Sudan
Source: BMC Res Notes. 2019 Apr 2;12:202. doi: 10.1186/s13104-019-4239-7 (PMC6446286; doi:10.1186/s13104-019-4239-7)
Supplement: Supplementary file 4 — Additional file 4: Table S2. Rai stage in age groups. [file 13104_2019_4239_MOESM4_ESM.docx]

Table S2: Rai stage in age groups

| Rai stage | ≤55 years n. (%) | >55 years n. (%) | n.% in total | P value* |
| --- | --- | --- | --- | --- |
| 0 | 1(3.2%) | 9 (11.4%) | 10(9.1%) | 0.629 |
| I | 5(16.1%) | 18(22.8%) | 23(20.9%) |  |
| II | 10(32.3%) | 13(16.5%) | 23(20.9%) |  |
| III | 11(35.5%) | 25(31.6%) | 36(32.7%) |  |
| IV | 4(12.9%) | 14(17.7%) | 18(16.4%) |  |

(n=110). *P value significant below 0.05 Kruskal-Wallis Test

Due to late presentation at diagnosis nearly half of patients were at Rai late stages. Elder patients had relatively higher percentage of Rai stage IV compared to young patients.
