# Supplementary material for: Clinical presentation and hematological profile among young and old chronic lymphocytic leukemia patients in Sudan
Source: BMC Res Notes. 2019 Apr 2;12:202. doi: 10.1186/s13104-019-4239-7 (PMC6446286; doi:10.1186/s13104-019-4239-7)
Supplement: Supplementary file 5 — Additional file 5: Table S3. Modified Rai stage in age groups. [file 13104_2019_4239_MOESM5_ESM.docx]

Table S3: Modified Rai stage in age groups

| Rai stage | ≤55 years n. (%) | >55 years n. (%) | n.% in total | P value* |
| --- | --- | --- | --- | --- |
| Low risk(0) | 1(3.2%) | 9 (11.4%) | 10(9.1%) | 0.767 |
| Intermediate(I,II) | 15(48.39%) | 31(39.23%) | 46(41.82%) |  |
| High risk (III,IV) | 15(48.39%) | 39(49.37%) | 54(49.1%) |  |

(n=110). *P value was significant below 0.05. Kruskal-Wallis Test

Ninety two percent of all patients presented at intermediate or high Rai stages with comparable distribution in both age groups.
