# Supplementary material for: Clinical presentation and hematological profile among young and old chronic lymphocytic leukemia patients in Sudan
Source: BMC Res Notes. 2019 Apr 2;12:202. doi: 10.1186/s13104-019-4239-7 (PMC6446286; doi:10.1186/s13104-019-4239-7)
Supplement: Supplementary file 6 — Additional file 6: Table S4. Binet stage in age groups. [file 13104_2019_4239_MOESM6_ESM.docx]

Table S4: Binet stage in age groups

| Binet stage | ≤55 years n. (%) | >55 years n. (%) | n.% in total | P value* |
| --- | --- | --- | --- | --- |
| A | 6(19.35%) | 27 (34.18%) | 33(30.00%) | 0.268 |
| B | 12(38.71%) | 23(29.11%) | 35(31.82%) |  |
| C | 13(41.94%) | 29(36.71%) | 42(38.18%) |  |

(n=110).*P value significant below 0.05 Kruskal-Wallis Test

About 70% of all patients presented at Binet B or C stages and young patient group recorder higher percentages in stages B and C comparing to elder patients.
