# Supplementary material for: Clinical presentation and hematological profile among young and old chronic lymphocytic leukemia patients in Sudan
Source: BMC Res Notes. 2019 Apr 2;12:202. doi: 10.1186/s13104-019-4239-7 (PMC6446286; doi:10.1186/s13104-019-4239-7)
Supplement: Supplementary file 7 — Additional file 7: Figure S3. Modified Rai risk staging with sex. [file 13104_2019_4239_MOESM7_ESM.docx]

Figure S3: Modified Rai risk staging with Sex (n=110).

Males had higher percentage of advanced modified Rai stages at presentation (intermediate and high risk) 95%, comparing to 80% of females presented at advanced modified Rai stages, but was not statistically significant.
