# Supplementary material for: Clinical presentation and hematological profile among young and old chronic lymphocytic leukemia patients in Sudan
Source: BMC Res Notes. 2019 Apr 2;12:202. doi: 10.1186/s13104-019-4239-7 (PMC6446286; doi:10.1186/s13104-019-4239-7)
Supplement: Supplementary file 8 — Additional file 8: Figure S4. Binet staging with sex. [file 13104_2019_4239_MOESM8_ESM.docx]

Figure S4: Binet staging with Sex (n=110).

Regarding Binet stage, (45%) of females were presented at Binet stage C, whereas in males (37%) were presented at Binet B stage, with no significant correlation.
