# Supplementary material for: Clinical presentation and hematological profile among young and old chronic lymphocytic leukemia patients in Sudan
Source: BMC Res Notes. 2019 Apr 2;12:202. doi: 10.1186/s13104-019-4239-7 (PMC6446286; doi:10.1186/s13104-019-4239-7)
Supplement: Supplementary file 9 — Additional file 9: Table S5. Expression of CD38 and ZAP70 according to age and sex groups. [file 13104_2019_4239_MOESM9_ESM.docx]

Table S5: Expression of CD38 and ZAP70 according to age and sex groups.

| Parameter | ≤55 years n=31 | >55 years n=79 | *P value | Male | Female | *P value |
| --- | --- | --- | --- | --- | --- | --- |
|  | N (%) | N (%) |  | N (%) | N (%) |  |
| ZAP-70+ | 11 (35.5%) | 25 (31.6%) | 0.432 | 26 (32.91%) | 10 (32.25%) | 0.568 |
| CD38+ | 14 (45.2%) | 27 (34.2%) | 0.196 | 31(39.24%) | 10 (32.25%) | 0.325 |

(n=110). *P value was significant below 0.05.Fischer exact test.
